# Case Management for Enhancing Wellbeing, Resilience, and Quality of Life in Caregivers of Children and Adolescents With Chronic Illnesses and Disabilities: A Systematic Review and Meta‐Analysis

**DOI:** 10.1111/nhs.70096

**Published:** 2025-05-08

**Authors:** Jan Broll, Sarah K. Schäfer, Jutta Stoffers‐Winterling, Sarah Hölzen, Isabella Helmreich, Klaus Lieb

**Affiliations:** ^1^ Leibniz Institute for Resilience Research (LIR) Mainz Germany; ^2^ Clinical Psychology, Psychotherapy and Psychodiagnostics Technical University of Braunschweig Braunschweig Germany; ^3^ Department of Psychiatry and Psychotherapy University Medical Center of Johannes Gutenberg University Mainz Mainz Germany

**Keywords:** caregivers, case management, chronically ill children, mental health, systematic review

## Abstract

Caregivers of children and adolescents with chronic illnesses often experience mental health challenges, which increase their risk of poor quality of life. This systematic review evaluates the effectiveness of case management interventions in improving caregivers' positive mental health, psychological distress, and satisfaction with health care services. We included (cluster) randomized controlled trials that evaluated the effects of case management. Systematic searches were conducted in PubMed, Scopus, CENTRAL, and PsycINFO up to 30 June 2024. Risk of bias was assessed using the Cochrane tool (RoB 2), and multi‐level meta‐analyses were performed for post‐intervention outcomes, while follow‐up data were synthesized qualitatively due to limited long‐term evidence. A total of 17 studies were included, providing multiple effect estimates for different types of outcomes, showing small positive effects of case management on mental health (analyses based on 8 studies), psychological distress (6 studies), and satisfaction with health services (9 studies). Although case management interventions show promise in supporting carers, the limited number of studies limits the strength and generalizability of the findings. Further research is needed to explore the long‐term effectiveness of such interventions.

**Trial Registration:** The review protocol was prospectively registered at PROSPERO (CRD4202453375).

**Summary**

- Case management interventions show small but meaningful improvements in caregivers' positive mental health, reductions in mental distress, and enhanced satisfaction with healthcare services.
- The current evidence base is limited by a small number of studies, particularly for follow‐up data, and variability in study quality and design.
- Further research is needed to confirm these findings, explore long‐term effects, and develop more targeted case management strategies for caregivers of chronically ill children.

## 1 Introduction

In recent decades, the prevalence of chronic health conditions among children and adolescents has increased significantly (Barrio Cortes et al. 2020; Compas et al. 2012; Davis et al. 2014). Chronic health conditions include both chronic diseases and chronic physical disabilities and are generally defined as conditions that persist for more than 12 months and are severe enough to limit activities of daily living. Examples of chronic conditions include cystic fibrosis, congenital heart disease, and diabetes mellitus (McEachern et al. 2023).

These health conditions can be so severe that children and adolescents require extensive care and support to manage their conditions effectively (McEachern et al. 2023). Typically, family members, most commonly mothers, take on the primary caregiving role for these children (Johnson and Onieka Mendoza 2018). These caregivers frequently encounter significant challenges and heightened levels of stress due to their caregiving responsibilities (Cousino and Hazen 2013; Law et al. 2019). These challenges include financial strains as well as emotional and physical exhaustion (Cousino and Hazen 2013). In general, caring for a child with a disability or chronic illness significantly increases the risk of health, social, and economic harm, often resulting in poorer living and care conditions for those families (Page et al. 2020).

The cumulative burden of caregiving severely compromises the wellbeing and quality of life of family carers (Cohn et al. 2020). This highlights the critical need for interventions to support caregivers' health and reduce their stress. Case management is a collaborative process that includes assessment, planning, coordination, and advocacy to help individuals and families access health care and support services (Mullahy 2016). This process is designed to improve communication among healthcare providers and ensure continuity in treatment plans, thereby reducing the fragmented and confusing care situations that many caregivers encounter (Boyden et al. 2022). Additionally, case management offers emotional support, counseling, and coping strategies to alleviate stress and anxiety associated with caregiving responsibilities (Bromer and Korfmacher 2017). By addressing both practical and emotional needs, case management has the potential to improve the wellbeing of families caring for children with disabilities or chronic health conditions.

Most reviews to date have examined the impact of case management on the well‐being and satisfaction with healthcare services of older adults (e.g., Berthelsen and Kristensson 2015; Corvol et al. 2017; Schiller et al. 2022). These studies generally found positive effects of case management interventions on caregivers' well‐being and increased use of and satisfaction with support services. However, the results were marked by heterogeneity, reflecting variations in study design and intervention approaches (Berthelsen and Kristensson 2015; Corvol et al. 2017; Pimouguet et al. 2010; Schiller et al. 2022; You et al. 2012). Only a few reviews have specifically focused on caregivers of children and adolescents with chronic health conditions. A review by Van Orne (2022) examined care coordination programs for children with medical complexity, noting varied strategies and inconsistent effects. Another review by Edelstein et al. (2017) on caregivers of children and young adults identified six intervention types for reducing caregiver stress: care coordination models, respite care, telemedicine, peer and emotional support, insurance and employment benefits, and health and related supports, which showed promising but inconclusive evidence of effectiveness.

To date, there are relatively few reviews that address caregivers of children and adolescents, and none of these specifically examine the effects of interventions on caregivers' mental health and overall wellbeing. With our review, we aim at addressing this gap. First, this review seeks to address the lack of research focus on caregivers of children and adolescents, an area often overshadowed by studies on adult caregiving. It is important to note that findings from research on caregivers for adults may not automatically translate to caregivers of children with chronic health conditions. The caregiving demands, support needs, and emotional challenges faced by these two groups may differ significantly. Second, the existing evidence is synthesized to help caregivers, health professionals, policymakers, and researchers understand the impact of case management in supporting parents and carers.

## 2 Materials and Methods

This systematic review adheres to the standards of the Cochrane Collaboration (Higgins et al. 2023) and is reported in accordance with the Preferred Reporting Items for Systematic Reviews and Meta‐Analyses (PRISMA; Page et al. 2021). This review was prospectively preregistered under PROSPERO preregistration‐ID: CRD42024533750.

### 2.1 Eligibility Criteria

Eligible studies were (cluster) randomized controlled trials ([c]RCTs) that examined case management interventions for caregivers of children and adolescents with chronic illnesses, disabilities, or other health‐related conditions requiring special care. Chronic conditions were defined as conditions that required ongoing medical attention or limited daily activities, such as asthma, diabetes, cystic fibrosis, epilepsy, Crohn's disease, juvenile arthritis, and sickle cell anemia. Disabilities included physical, intellectual, or developmental impairments that substantially affected major life activities (Bernell and Howard 2016). The interventions targeted individuals primarily responsible for providing care to children and adolescents with long‐term health needs at home. Case management was defined as interventions that aimed to support caregivers in coordinating care, which mentioned case management as a relevant theoretical framework, or addressed concepts of care coordination and support as primary or secondary outcomes. The interventions themselves could be delivered in any setting, including clinics, study centers, or family homes, and were eligible regardless of their delivery mode (face‐to‐face or digital), content, or duration. All types of comparators were eligible, including waitlist controls, care as usual, attention controls, and active controls. Eligible studies were required to assess at least one outcome in caregivers related to mental distress (e.g., anxiety, depression, general distress, posttraumatic stress, symptoms of stress), positive mental health (e.g., wellbeing, life satisfaction), or healthcare utilization (e.g., access to therapy services, frequency of doctor or specialist visits, reduction in emergency visits).

Studies were excluded if they did not include carers of children or young people or if they focused on carers of other populations, such as older adults (e.g., carers of people with dementia). Studies were also excluded if they were not randomized controlled trials ([c]RCTs), did not evaluate case management as a central intervention, or did not report caregiver‐specific outcomes related to mental distress, positive mental health, or healthcare utilization. Further exclusion criteria applied to studies focusing on caregivers of children and adolescents with mental health conditions as defined by the International Classification of Diseases, 11th Revision (ICD‐11), or those conducted exclusively in clinical or institutional settings.

### 2.2 Search Strategy

Searches were performed on June 30, 2024, in the Cochrane Central Register of Controlled Trials (CENTRAL), Pubmed, PsycINFO, and PsycArticles via EbscoHost, Scopus, and Web of Science. Search terms comprised five clusters that were searched in titles, abstracts, and keywords: terms related to (i) caregivers (e.g., “caregiver” or “guardian”), (ii) children and adolescents (e.g., “child” or “minor”), (iii) home nursing (e.g., “home nursing” or “long term care”), (iv) case management (e.g., “case management” or “care program”), and (v) study design (e.g., “randomized controlled trial” or “controlled clinical trial”). Terms within one cluster were linked using the Boolean operator OR, while clusters were linked using the operator AND. If applicable, we used Medical Subject Headings (MeSH). Full search strategies per database are presented in Data S1. Moreover, we checked the reference lists of included studies for additional potentially eligible primary studies.

### 2.3 Study Selection

Deduplication was performed in Zotero (2024). Records from the database searches were independently assessed by two reviewers (JB, SH), one holding a Master's degree (JB) and the other a Bachelor's degree (SH), at both the title/abstract and full‐text levels using Rayyan (Ouzzani et al. 2016). Cohen's kappa (Cohen 1960) as a measurement for inter‐rater reliability was 0.78 for the title/abstract screening and 0.80 for the full‐text screening. Disagreements were resolved through discussion or by consulting a third reviewer (SKS, KL).

### 2.4 Data Extraction

We designed a tailored data extraction form for this review. We designed a tailored data extraction sheet for this review. Two reviewers (JB, SH), independently extracted descriptive data and quantitative outcomes from eligible primary studies to ensure accuracy and minimize biases. Discrepancies between reviewers were resolved through discussion or consultation with a third reviewer (SKS, KL). When data necessary for calculating effect estimates were missing or unclear, we contacted the corresponding authors of the primary studies via email. Reminder emails were sent after 14 days if no response was received. If no clarification was obtained, the respective studies were excluded from the review due to insufficient data. No automation tools were used as part of the data extraction process.

### 2.5 Risk of Bias Assessment

Two team members (JB, SH) independently evaluated the risk of bias in the included primary studies using the Cochrane risk‐of‐bias tool for randomized trials (RoB2; Sterne et al. 2019). The RoB2 tool assesses bias from the following domains: (i) randomization process, (ii) deviations from the intended intervention, (iii) missing outcome data, (iv) outcome measurement, and (v) selection of reported outcomes. Bias assessments were conducted at the levels of individual outcomes and overall study, with judgments categorized as “low risk,” “high risk,” or “some concerns.”

We evaluated potential publication bias through visual inspection of contour‐enhanced funnel plots (Peters et al. 2008) and statistically by means of rank correlation tests (Begg and Mazumdar 1994).

### 2.6 Data Synthesis

Included studies were summarized both narratively and in tabular form. Descriptive methods were used to summarize the demographic variables and characteristics of the studies. Pairwise meta‐analyses were conducted for primary outcomes only when at least five studies were available per comparison and outcome, ensuring sufficient data for meaningful analysis (Pigott 2012). For outcomes with fewer than five studies, findings were summarized narratively (Barnett‐Page and Thomas 2009). Some studies had more than one intervention group. For those studies, we averaged the results across groups that employed case management interventions. In cases where data necessary for the calculation of effect estimates were missing or unclear, we contacted the corresponding authors of the primary studies via e‐mail. After 14 days, a reminder e‐mail was sent if no response was received. Some studies also reported effects on children and adolescents. However, as the primary focus of this review is on caregivers, these findings were briefly summarized rather than analyzed in detail.

Meta‐analyses were performed in R version 4.4.1 (R Core Team 2024), using the packages *tidyverse* (Wickham et al. 2019), *easystats* (Lüdecke et al. 2022), *esc* (Lüdecke 2024), *metafor* (Viechtbauer 2010), and *robvis* (McGuinness 2019). Standardized mean differences (SMDs, Hedges' *g*) at the post‐intervention assessments were calculated as effect estimates, with their 95% confidence intervals (CIs) as indicators of statistical significance. SMDs were calculated based on means (*M*s) and standard deviations (SDs) at post‐intervention assessments. Positive SMDs indicated favorable intervention effects for positive mental health outcomes and satisfaction with healthcare services, whereas negative SMDs indicated favorable intervention effects for mental distress. To account for the uncertainty of meta‐analytic results, we calculated 95% prediction intervals (PIs) as an estimate of the range in which 95% of future observations are expected to fall when more than 10 effect estimates were available (Higgins et al. 2023).

The main analyses aimed to assess whether case management interventions influenced mental distress, positive mental health, and healthcare utilization. Three multilevel meta‐analysis models were used, each focusing on one of the primary outcomes. The multilevel approach was used to account for the possibility that a single study might report multiple relevant outcomes (e.g., well‐being and family quality of life). The models weighted effect estimates by their respective variances and accounted for variability between studies by including a random effect at the study level. Restricted Maximum Likelihood (REML) estimations were applied, and cluster‐robust tests with confidence intervals (CIs) were reported. Statistical heterogeneity was evaluated using Cochran's *Q* (Cochran 1954) and the *I* ^2^ statistic (Higgins et al. 2023), with values above 50% indicating substantial heterogeneity. The certainty of evidence was assessed using the Grading of Recommendations, Assessment, Development and Evaluations (GRADE; Schünemann 2022) approach.

Moderator analyses were conducted to explore whether the effectiveness of case management interventions varied depending on specific factors. The moderators tested included gender, intervention setting (e.g., clinic‐based vs. home‐based), intervention mode (e.g., in‐person vs. digital), and intervention duration. These analyses were performed for the primary outcomes of positive mental health, mental distress, and satisfaction with healthcare services. Random‐effects meta‐regression models were used for each outcome, with effect estimates as the dependent variable and moderators as independent variables. These models accounted for between‐study variability, and omnibus tests were used to determine whether each moderator explained a significant proportion of heterogeneity. All analyses were performed using robust variance estimation to handle dependent effect estimates within studies.

## 3 Results

### 3.1 Search Outcomes

**FIGURE 1:**
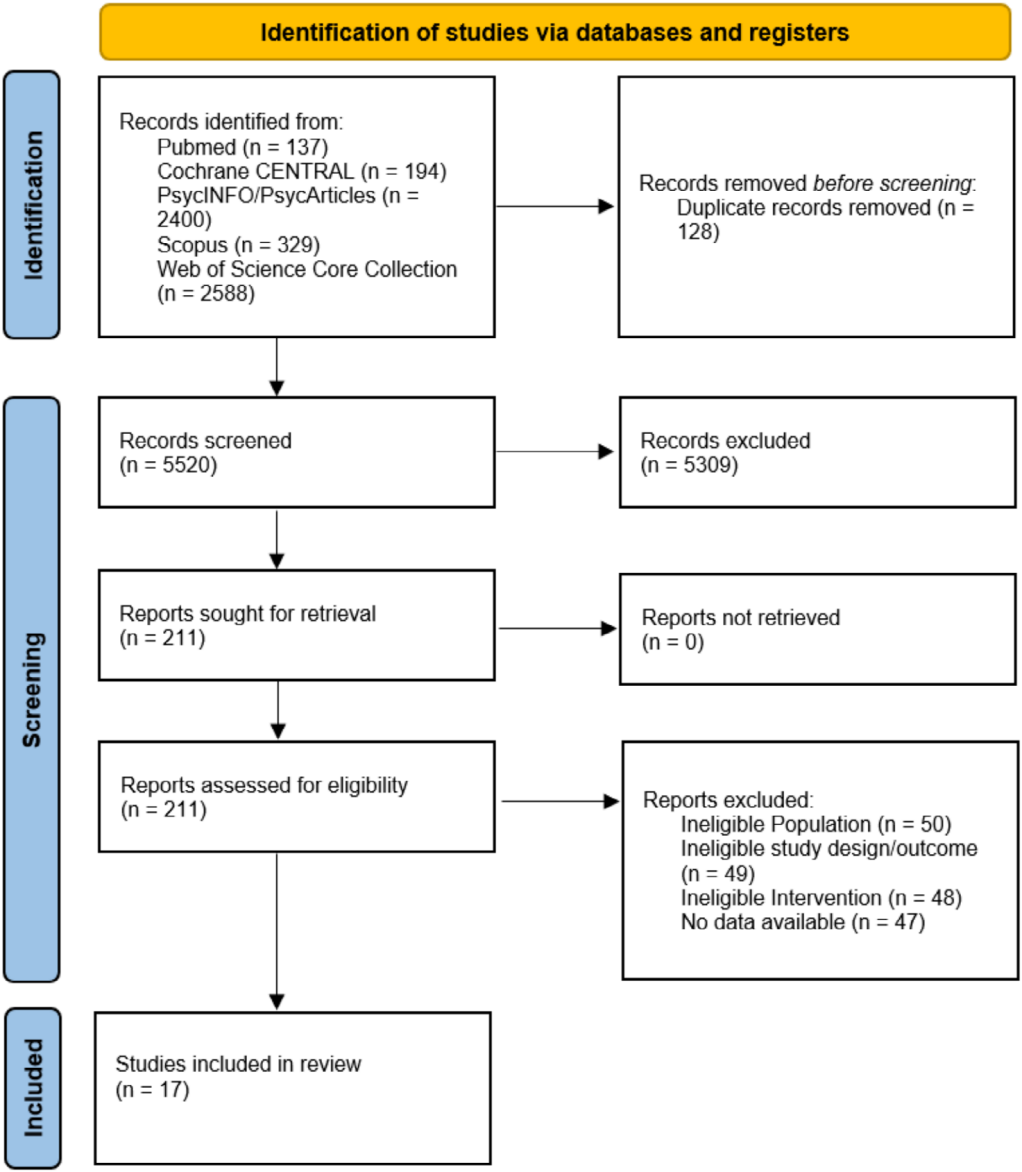
PRISMA flowchart. Flowchart according to the preferred reporting items for systematic reviews and meta‐analyses. *n*, number of studies/records/reports.

Our search of electronic databases initially yielded 5648 records. After removing 128 duplicates, 5520 unique records remained. These were screened at the title and abstract level, and 211 records were assessed at the full‐text level, of which 17 studies met the inclusion criteria (see Figure 1). All 17 studies included after the full‐text screening were also included in the meta‐analysis.

### 3.2 Study Characteristics

The studies included in this review were conducted across diverse geographical settings, primarily in high‐income countries. Specifically, 11 studies were from the United States, two from China, and one each from Australia, Canada, Taiwan, and Turkey. Most studies (84%) originated in high‐income countries, with only 16% representing middle‐income countries, reflecting a scarcity of data from low‐resource settings. All 17 studies employed randomized controlled trial (RCT) designs, ensuring a high level of methodological rigor in evaluating the effectiveness of case management interventions for caregivers.

Table 1 details the characteristics of the 17 primary studies included in the analysis, which comprised a total of 1923 caregivers, of whom 1137 (60%) were women, with not all studies reporting on gender. The mean age of caregivers was 32.75 years (SD = 7.92), and the mean age of children was 7.40 years (SD = 3.52).

**TABLE 1:** Characteristics of included studies.

Study ID	Country	Population [type; age (*M* ± SD); % women]	*n*_randomized_; *n*_IG_; *n*_CG_	Intervention	Theoretical basis	Setting	Delivery	Intensity of intervention	Comparator	Outcomes eligible for review
Badr et al. (2006)	USA	Mothers of infants with suspected brain injury; 27.31 ± 2.16; 100%	62; IG: 32; CG: 30	Curriculum and Monitoring System (CAMS)	Not specified	At home	In person	48 weeks	Treatment‐As‐Usual	Parenting stress; cognitive and motor development
Bernie et al. (2023)	Australia	Families waiting for autism assessment; nr; 75%	16; IG: 9; CG: 7	Occupational performance coaching combined with service navigation	Not specified	At home	In person, video	4 × 30–70 Minutes	Treatment‐As‐Usual	Functional goal attainment, parenting stress, family quality of life
Bilgin and Gozum (2009)	Turkey	Mothers of children with intellectual disabilities; 34.06 years ± 7.36; 100%	90; IG: 45; CG: 45	Education program on burnout	Burnout theory	Private education and rehabilitation centers	In person	5 × 2 weeks; 60 min	Waitlist	Burnout
Burton et al. (2018)	USA	Families with children with developmental disability, nr; 99%	87; IG: 41; CG: 46	Nurturing program for parents and their children with special needs and health challenges	Not specified	Clinic	In Person, video	1 × 12 weeks; 120 min	Treatment‐As‐Usual	Child expectation, empathy toward children, family empowerment
Cady et al. (2015)	USA	Children with medical complexity; nr; nr	90; IG: 59; CG: 31	Telehealth care coordination	Presler'smodel of clinic‐based care coordination	At home	Telephone, video	96 weeks	Treatment‐As‐Usual	Need for care coordination; adequacy of care coordination
Chan et al. (2007)	USA	Parents of children with persistent asthma, nr; nr	87; IG: 60; CG: 60	Virtual asthma education and case management	Not specified	At home	In person, online	6 sessions within 52 weeks	Treatment‐As‐Usual	Quality of life
Chen et al. (2013)	Taiwan	Caregivers of children with asthma, 37.80 years ± 7.01; 86%	65; IG: 33; CG: 32	Self‐management interactive support	Not specified	At home, clinic	Household and clinic	16 sessions within 52 weeks	Treatment‐As‐Usual	Asthma knowledge, asthma attitude
Cohen et al. (2023)	Canada	Parent with children with medical complexity; nr; 84%	139; IG: 77; CG: 62	Complex care for kids Ontario	Not specified	Clinic	In person	96 weeks	Waitlist	Satisfaction with coordination of care among health care professionals, Satisfaction with coordination of care between health care professionals and families, utility of care planning tools, physical health, mental health, fatigue, sleep disturbance, satisfaction with life, healthcare service
Farmer et al. (2011)	USA	Parents children with special health care needs; nr; 66%	100; IG: 50; CG: 50	Care coordination intervention	Not specified	At home	In person, telephone	24 weeks	Waitlist	Satisfaction with health services, family functioning, child functioning
Flores et al. (2009)	USA	African‐American or Latino parents of asthmatic children; 31.88 years ± 8.37, 91%	220; IG: 112; CG: 108	Parent mentors	Not specified	At home	In person, telephone	48 weeks	Treatment‐As‐Usual	Parent‐reported health‐related quality of life, asthma caregiver's quality of life
Liu et al. (2020)	China	Caregivers of children with epilepsy; 28.87 years ± 5.48, 62%	108; IG: 58; CG: 50	Home self‐management	Not specified	At home, clinic	In person, online, telephone	72 weeks; 1 × week	Treatment‐As‐Usual	Family quality of life: family interaction, parenting, emotional well‐being, physical/material well‐being, disability‐related support
Looman et al. (2015)	USA	Caregivers of children with medical complexity; nr; 67%	163; IG: 108; CG: 55	Telehealth care	Not specified	At home	Telephone, video	96 weeks	Treatment‐As‐Usual	Perceptions of health care, provider communication
Looman et al. (2018)	USA	Caregivers of children with medical complexity; nr; 67%	163; IG: 108; CG: 55	Telehealth care	Not specified	At home	Telephone, video	96 weeks	Treatment‐As‐Usual	Family impact
Moody et al. (2019)	USA	Parents of children with autism; 34.27 years ± 5.75, 90%	67; IG: 33; CG: 34	Colorado parent mentoring program	Not specified	At home, center	In person	24 weeks	Waitlist	Family support
Seid et al. (2010)	USA	Families of children with persistent asthma; nr; nr	252; IG: 165; CG: 84	Care coordination + problem‐solving skills training	Not specified	At home	In person	6 weeks	Waitlist	Quality of life
Thompkins et al. (2021)	USA	Adolescents with cancer and their families; 45.90 years ± 8.20, 82%	126; IG: 83; CG: 43	Family‐centered advance care planning for teens with cancer (face‐tc)	Not specified	Clinic	In person	3 weeks	Treatment‐As‐Usual	Caregiver strain, caregiver distress, family well‐being, positive caregiving appraisal
Zhang et al. (2023)	China	Infants after congenital heart surgery and their parents; 30.10 years ± 4.00; nr	88; IG: 44; CG: 44	Telemedicine	Not specified	At home	Video	12 weeks	Control	Parenting burden, caregiver knowledge, caregiver competence

The studies included caregivers of children and adolescents with chronic and complex health conditions. Of the included studies, 47% focused on children with complex medical problems such as asthma, epilepsy, heart disease, cancer, and other chronic illnesses. Neurological and developmental conditions were the focus of 35% of the studies, addressing conditions such as brain injury, autism spectrum disorder (ASD), intellectual disabilities, and other developmental conditions. An additional 18% of the studies involved children with mental and physical disabilities requiring specialized healthcare.

The interventions were case management interventions aimed at supporting caregivers. These interventions were mostly delivered in family homes (59%), with others being conducted in clinical or research settings (24%) or a mix of both (17%). All interventions were face‐to‐face, often supplemented by telephone (29%) and online/video (23%) support programs. Most interventions were guided by professionals such as nurses, therapists, or social workers (89%), while only 11% did not involve professional contact, such as peer support groups. The average duration of the interventions was 44.2 weeks (SD = 35.78). Three studies (24%) reported follow‐up data between 3 and 24 months. All studies employed passive comparators, such as waitlist controls or care‐as‐usual conditions.

The content of the interventions varied. Some focused on care coordination (e.g., Cohen et al. 2023; Farmer et al. 2011; Seid et al. 2010), while others incorporated education and self‐management training (e.g., Chen et al. 2013; Liu et al. 2020). A few studies used mentoring programs (e.g., Flores et al. 2009; Moody et al. 2019) or combined case management with psychosocial support (e.g., Bernie et al. 2023; Thompkins et al. 2021). While these case management interventions varied in structure and delivery, most studies did not explicitly ground their intervention designs in established psychological theories. Few studies referenced theoretical models, such as burnout theory (Maslach 1999), to explain the mechanisms through which case management may improve caregiver outcomes.

The outcomes measured included positive mental health, mental distress, and healthcare utilization. Most outcomes were in the satisfaction with healthcare services category (40%), followed by positive mental health (32%), and mental distress (28%). Examples of measures of positive mental health included “life satisfaction,” “emotional well‐being,” and “physical health.” Examples of satisfaction with healthcare services included “communication with providers,” “satisfaction with care coordination,” and “care coordination adequacy”. Measures for mental distress focused on aspects such as “parental stress,” “emotional burnout,” and “caregiver distress” (see Table 2 for a detailed overview).

**TABLE 2:** Results of main analyses for primary outcomes comparing case management interventions for caregivers and comparators.

	*n*	*k*	SMDs	95% CI	95% PI	*p*	*Q*	df	*p*(*Q*)	*I* ^2^
*Positive mental health*	8	19	0.25	[0.07, 0.32]	[0.12, 0.38]	< 0.001	18.40	22	0.683	10.8%
*Mental distress*	6	16	−0.29	[−0.53, −0.04]	[−0.53, −0.03]	0.024	15.51	15	0.953	2.57%
*Satisfaction with healthcare services*	9	23	0.25	[0.12, 0.38]	[0.070, 0.43]	< 0.001	18.44	24	0.682	7.21%

*Note:* The multilevel meta‐analysis on positive mental health indicators included well‐being, mental health, empathy, autonomy, and caregiver appraisal. Mental distress comprised measures of burden, burnout, cognitive impact, physical impact, emotional impact, social impact, stress, mental health, and worry. Satisfaction with healthcare services comprised measures of satisfaction with help services, use of help services, and self‐efficacy toward these services. For positive mental health and resilience factors, positive SMDs indicate favorable intervention effects (i.e., higher levels of positive mental health in the intervention group compared with the control group). For distress indicators, negative SMDs indicate favorable effects of the Intervention (i.e., lower distress in the intervention group compared with the control group). For Satisfaction with healthcare services, positive SMDs indicate favorable intervention effects (i.e., higher satisfaction with care in the intervention group compared with the control group). All tests and reported statistics use cluster‐robust estimates to account for non‐independent effect estimates within studies.

Abbreviations: 95% CI, 95% confidence interval; 95% PI, 95% prediction interval; df, degrees of freedom; *I* ^2^, heterogeneity index in percentage (range: 0%–100%); *k*, number of effect estimates; *n*, number of studies; *Q*, Cochran's *Q* statistic with *p* value; SMD, standardized mean difference.

In addition to caregiver outcomes, seven studies (41%) reported on outcomes for the children being cared for. Most of these outcomes were measures of the children's positive mental health, such as well‐being, quality of life, or mental health.

### 3.3 Risk of Bias

**FIGURE 2:**
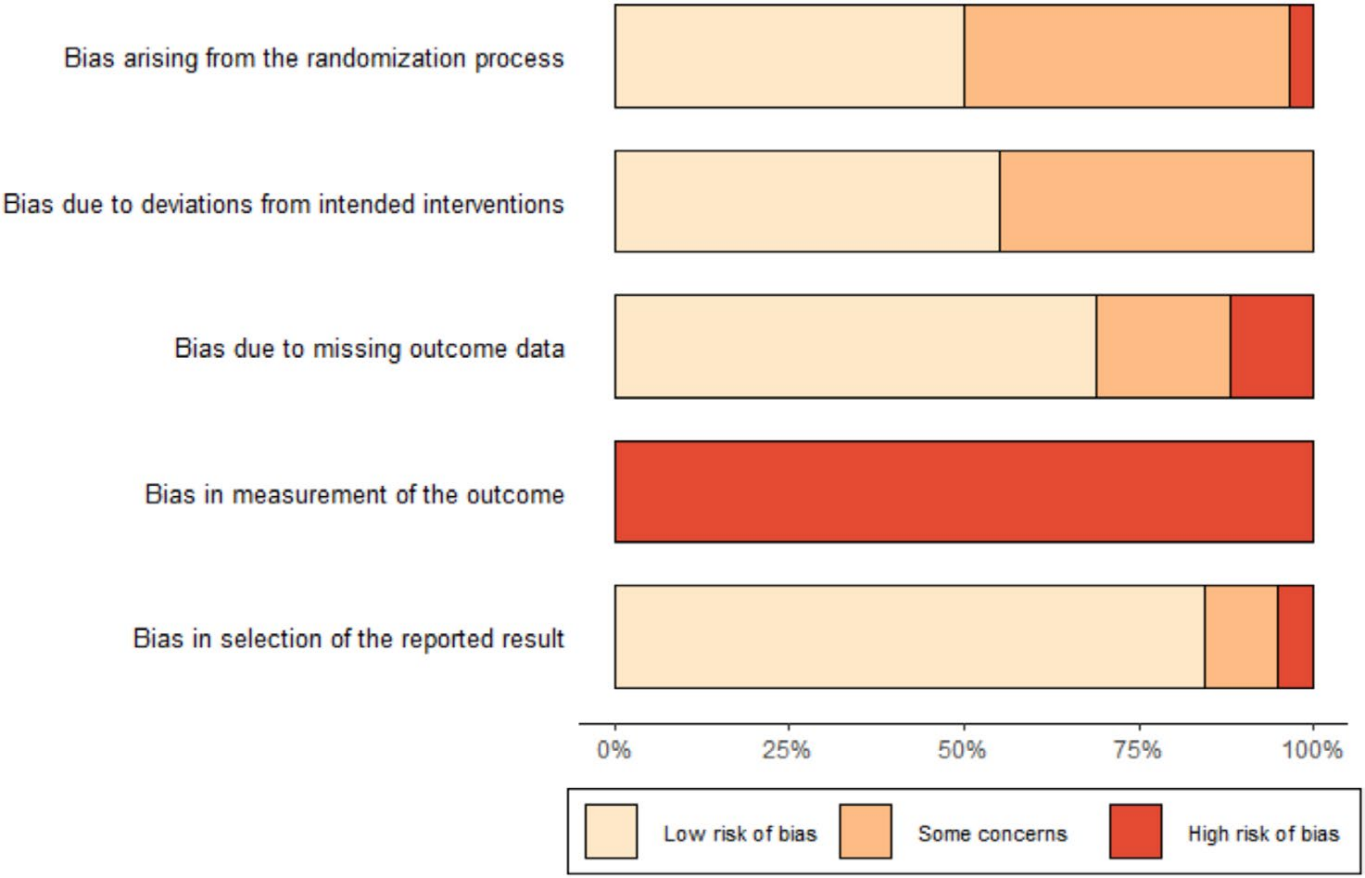
Risk of bias across effect estimates at post‐intervention. Risk of bias in percentages across effect estimates assessed using the Cochrane risk‐of‐bias tool for randomized trials (RoB2).

There was a high risk of bias (see Figure 2). The greatest risks were found with regard to outcome measurement (100% high risk). The second highest risk was found regarding the randomization process (47% moderate risk, 3.4% high risk) and then deviations from intervention (45% moderate risk). There were fewer methodological flaws in the other domains: missing outcome (19% moderate risk, 12% high risk) and selection of reported results (10% moderate risk, 5% high risk).

**FIGURE 3:**
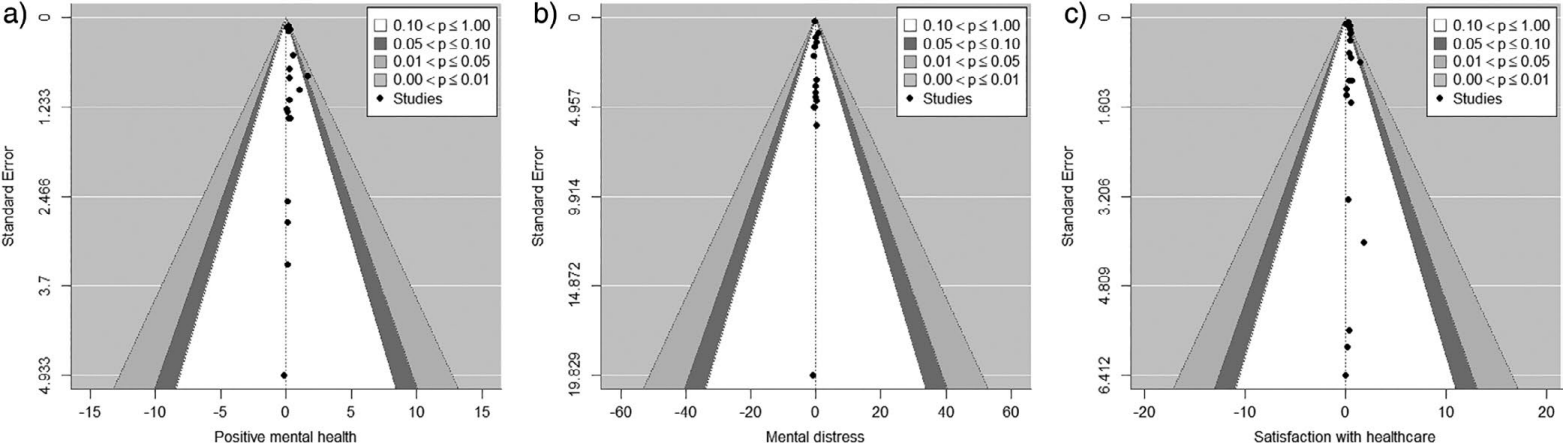
Contour‐enhanced meta‐analysis funnel plots for (a) positive mental health; (b) mental distress; and (c) satisfaction with health care service.

The funnel plot for positive mental health was largely symmetrical (see Figure 3).

**FIGURE 4:**
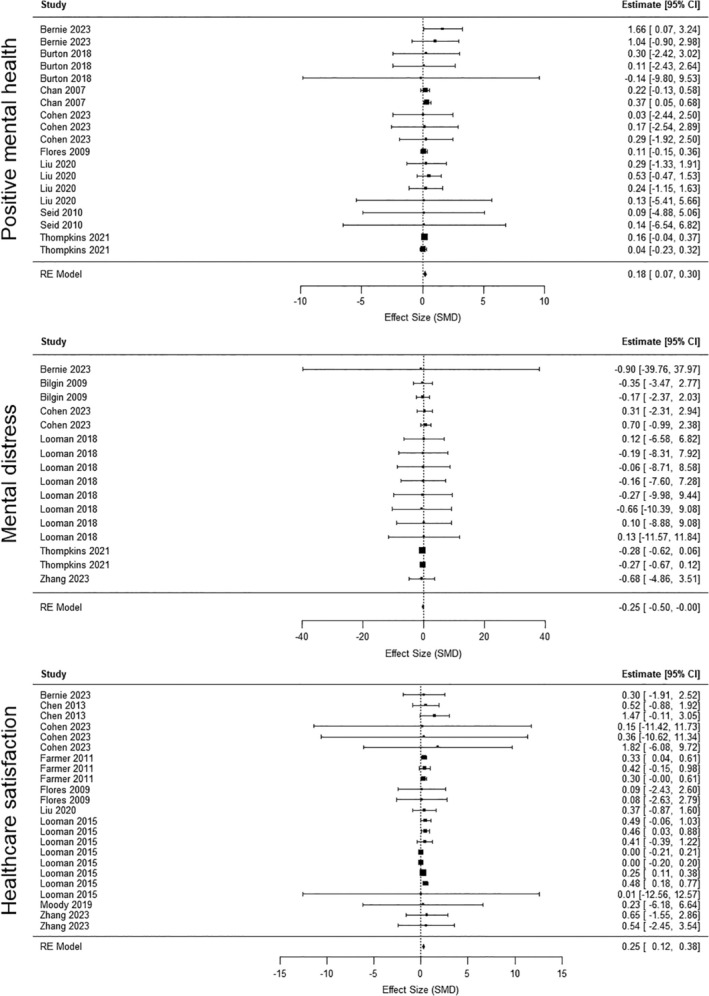
Forest plots for the analyses on positive mental health, mental distress, and health care utilization.

The rank correlation test for funnel plot asymmetry did not yield a significant result (Kendall's tau = −0.16, *p* = 0.369), indicating no evidence of asymmetry. The funnel plot for mental distress (see Figure 4) was also symmetrical and the rank correlation test provided no evidence for asymmetry (Kendall's tau = 0.05, *p* = 0.858). The funnel plot for satisfaction with healthcare services was slightly asymmetrical, also evidenced by a significant rank correlation test (Kendall's tau = −0.34, *p* = 0.029) (see Figure 4). Yet, further inspection did not suggest a disproportional amount of effect estimates falling into border significance areas of the funnel plot, suggesting no major impact of a publication bias on our findings.

### 3.4 Main Findings

#### 3.4.1 Intervention Effects at Post‐Intervention Assessment

##### 3.4.1.1 Positive Mental Health

Out of the 17 studies included in the systematic review, eight studies provided sufficient quantitative data for inclusion in the meta‐analysis, contributing a total of 23 effect estimates. The meta‐analysis revealed a small favorable effect of case management interventions over control conditions on positive mental health (SMD = 0.23, 95% CI [0.12, 0.38]). Heterogeneity was very low (*Q*(22) = 18.39, *p* = 0.683, *I* ^2^ = 10.8%).

##### 3.4.1.2 Mental Distress

Six studies (16 effect estimates) were included in the meta‐analysis for mental distress outcomes. The results showed a small to moderate favorable effect of case management interventions compared with control conditions (SMD = −0.29, 95% CI [−0.53, −0.04]). Heterogeneity was minimal (*Q*(15) = 15.51, *p* = 0.953, *I* ^2^ = 2.7%).

##### 3.4.1.3 Satisfaction With Healthcare Services

Nine studies (23 effect estimates) contributed data to the meta‐analysis of satisfaction with healthcare services. A small favorable effect of case management interventions was observed (SMD = 0.25, 95% CI [0.12, 0.38]). There was no substantial heterogeneity (*Q*(22) = 18.40, *p* = 0.683, *I* ^2^ = 8.06%).

The effects for all three outcomes—positive mental health, mental distress, and satisfaction with healthcare services—are illustrated in Figure 4, which presents forest plots summarizing the standardized mean differences and confidence intervals.

### 3.5 Intervention Effects at Follow‐Up Assessment

Three studies reported follow‐up data, which was insufficient to conduct a meta‐analysis. As a result, these studies were summarized narratively. Follow‐up measurements took place 3–24 months after the start of the intervention. Two studies, with a total of five effect estimates, reported follow‐up measurements of positive mental health. The follow‐up effect estimates for positive mental health ranged from 0.07, 95% CI [−2.13, 2.29] to 0.22, 95% CI [−4.75, 5.19], reflecting small positive improvements over time. Mental distress was reported in one study with two effect estimates. The follow‐up effect estimates for mental distress ranged from −0.25, 95% CI [−2.87, 2.37] to −0.45, 95% CI [−2.14, 1.23], indicating small to moderate reductions in distress over time. Two studies, with a total of 11 effect estimates, reported follow‐up measurements for satisfaction with healthcare services. The follow‐up effect estimates ranged from 0.05, 95% CI [−2.52, 2.61] to 0.50, 95% CI [0.36, 0.63], showing small to moderate positive effects.

### 3.6 Moderator Analysis

As moderator analyses were a priori planned, analyses on gender, intervention setting, intervention mode, and intervention duration were performed for all outcome types, although we found only minor evidence for heterogeneity (see Table 3).

**TABLE 3:** Results of moderator analyses at post‐intervention assessment.

	Positive mental health			Mental distress			Satisfaction with healthcare services		
	*n*/*k*	*M* (SMD) [95% CI]	*p*	*n*/*k*	*M* (SMD) [95% CI]	*p*	*n*/*k*	*M* (SMD) [95% CI]	*p*
Sociodemographic characteristics									
Gender (female)	8/19	*QM*(1) = 1.048	0.301	16/6	*QM*(1) = 0.02	0.866	23/9	*QM*(1) = 0.63	0.483
Intervention setting (at home vs. clinic/center vs. at home and clinic/center combined)									
Omnibus moderator test	8/19	*QM*(2) = 0.99	0.601	16/6	*QM*(2) = 0.01	0.914	23/9	*QM*(2) = 1.345	0.510
Delivery format (in person vs. in person/online vs. in person/video)									
Omnibus moderator test	8/9	*QM(1) =* 1.82	0.177	16/6	*QM*(1) = 0.01	0.967	23/9	*QM*(2) = 2.01	0.368
Intervention intensity in weeks									
Omnibus moderator test	18/9	*QM*(1) = 0.90	0.343	16/6	*QM*(1) = 1.21	0.272	23/9	*QM(*1) = 1.85	0.174

*Note:* *QM*(df) omnibus test for moderators, which follows approximately a *χ* ^2^ distribution, df is the degrees of freedom, *k* is the number of effect estimates.

None of these moderators showed a significant effect for any of the outcomes, suggesting that the impact of case management interventions was not meaningfully influenced by these factors in the included studies.

### 3.7 Effects on Children and Adolescents

Five studies, with a total of nine effect estimates, reported outcomes of positive mental health in children. The effect estimates ranged from −0.02, 95% CI [−8.95, 8.91] to 0.41, 95% CI [−2.88, 3.70], with most values indicating small to moderate favorable effects of case management over control conditions.

## 4 Discussion

This systematic review and meta‐analysis aimed to evaluate the effectiveness of case management interventions for caregivers of children with chronic health conditions. The post‐intervention results suggest that these interventions can lead to small but meaningful improvements in caregivers' well‐being and small to moderate reductions in mental distress as well as small improvements in satisfaction with healthcare services. These findings suggest that case management can be helpful in supporting caregivers by addressing their emotional and psychological distress (Cousino and Hazen 2013; Vonneilich et al. 2016). However, the overall impact of these interventions varies across outcomes. One notable limitation is that many interventions lacked a clear theoretical foundation, which limits the understanding of the mechanisms driving their effectiveness. Future studies should consider incorporating established psychological frameworks, such as stress‐coping models (Lai and Oei 2014), to better explain how and why case management interventions may influence caregiver well‐being. For instance, applying Lazarus and Folkman's Transactional Model of Stress and Coping (Lazarus and Folkman 1984) could help clarify whether case management reduces caregivers' perceived stress by enhancing their appraisal of available resources and improving their coping strategies.

The positive mental health outcomes at post‐intervention assessments demonstrated small but consistent favorable effects immediately after the end of the intervention. The low heterogeneity observed in these analyses suggests that case management interventions were similarly effective across settings, caregiver populations, and intervention types. The consistency across studies highlights the potential robustness of these interventions in improving caregivers' well‐being, which is often compromised due to the emotional and physical demands of managing chronic health conditions in children (Cohn et al. 2020). Importantly, these small effects should not be underestimated, as even marginal improvements in well‐being may have a cumulative impact over time (Fredrickson 2001). The small effect size suggests that while case managements interventions are beneficial, they may need to be part of broader support strategies to make substantial and sustainable differences in caregivers' quality of life (Edelstein et al. 2017). For example, integrating case management with other psychosocial interventions, such as cognitive–behavioral therapy (Kwon et al. 2017) or peer support programs (Joo et al. 2022), may enhance its impact on caregivers' mental health. Nevertheless, even small improvements can be meaningful for carers, who often experience lower levels of well‐being than the general population (Cousino and Hazen 2013; Page et al. 2020).

It is important to note that the findings are based on only eight studies, which represent a relatively small evidence base. Therefore, caution is needed when generalizing these results. Follow‐up analyses extended these findings, showing modest improvements in positive mental health up to 24 months after the intervention. While these results are encouraging and suggest potential long‐term benefits of case management interventions, the significance of these findings is very limited due to the small number of studies reporting follow‐up outcomes.

In addition to the positive mental health outcomes, the meta‐analysis on mental distress revealed similarly small to moderate favorable effects. This indicates that case management could play a meaningful role in reducing the emotional burden caregivers face. This is important as caregivers are often under increased stress due to the demanding caregiving situations at home (Cuzzocrea et al. 2016; Guite et al. 2018; Montirosso et al. 2021). From a psychological perspective, it is plausible that case management mitigates feelings of helplessness and perceived overload by offering structured guidance and emotional support, which in turn may reduce symptoms of anxiety or depression (Del‐Pino‐Casado et al. 2021). However, the number of included studies for mental distress was also low, and these results should therefore be interpreted with caution. Given the relatively limited scope of the studies included, further research is needed to confirm these findings. Our narrative synthesis on follow‐up assessments suggested that those favorable effects may remain stable over time, yet further studies on short‐ and long‐term effects are needed to allow for valid conclusions. Follow‐up analyses also indicated modest reductions in mental distress up to 24 months post‐intervention. These findings are promising and suggest that case management interventions may offer sustained relief for caregivers over time. However, the small number of studies reporting follow‐up outcomes limits the reliability and generalizability of these results. One possible explanation for these effects is that case management may alleviate mental distress by reducing caregivers' uncertainty in navigating healthcare services and improving their health literacy (Fields et al. 2018; Nickel et al. 2024).

Regarding satisfaction with healthcare services, case management interventions had a small positive impact, suggesting that these programs may improve caregivers' experiences in navigating and accessing care. By streamlining communication and coordination among healthcare providers, these interventions can reduce barriers to service delivery, potentially leading to higher satisfaction. However, again, the number of studies included in this analysis was small, with only nine studies reporting on healthcare utilization outcomes. Furthermore, the utilization of and the satisfaction with healthcare services is strongly influenced by the structure and accessibility of the healthcare system in each respective country, which further limits the generalizability of the findings. However, even small improvements in satisfaction with healthcare services can make a difference for caregivers by reducing the time and stress associated with finding and coordinating appropriate care (Coyne et al. 2019; Vonneilich et al. 2016). Follow‐up analyses suggested small improvements in satisfaction with healthcare services up to 24 months, but the limited number of studies restricts the strength and generalizability of these findings, similar to the results for positive mental health and mental distress. Interventions that specifically address gaps in care coordination—such as those that facilitate communication between specialists and primary care providers—may have a greater impact on caregiver satisfaction by ensuring a more seamless healthcare experience (Van Orne 2022; Yuen et al. 2024) Future research should explore whether caregivers in certain healthcare settings—such as publicly funded versus privately funded systems—experience different levels of improvement in satisfaction as a result of case management interventions. It would also be valuable to examine whether improved satisfaction mediates the relationship between case management and caregiver mental health, which would help clarify the pathways through which these interventions exert their effects.

The moderator analyses aimed to investigate whether factors such as gender, intervention setting (e.g., clinic‐based vs. home‐based), intervention mode (e.g., in‐person vs. digital), and intervention duration were associated with the effects of case management interventions. The results indicated that none of these variables had a significant moderating effect for any of the outcomes. This is likely due to the small heterogeneity observed in the analyses, which suggests that the effects of case management interventions were relatively consistent across different delivery formats, participant demographics, and intervention durations. However, the lack of significant findings may also reflect limitations of the included studies, such as the small between‐study differences for moderator variables. Further research with larger, more diverse datasets is needed to explore whether these factors might influence the effects of case management interventions.

Only a few studies reported outcomes related to children, and not all of these outcomes fitted within the scope of this review, resulting in the consideration of a small number of effect estimates. The effects on children's well‐being were small and inconsistent. While slight improvements were noted, further research is needed to better understand how these interventions might directly benefit children, particularly in terms of their development and well‐being. One possible mechanism is that improvements in caregiver mental health may have downstream positive effects on children's psychological adjustment, as parental stress and well‐being are closely linked to child outcomes (Neece et al. 2012). Future studies should examine this potential spillover effect to better understand the broader family‐level benefits of case management (Newland 2015). Such studies may also allow for the examination of the relationship between intervention effects of caregivers and their children.

### 4.1 Strength and Limitations

This review has several strengths most notably that it is the first to comprehensively examine case management interventions for caregivers of children with chronic health conditions. The findings provide valuable insights that may help to refine and improve interventions. However, there are also several limitations.

First, the small number of studies, particularly those reporting follow‐up assessments and outcomes for children, constrains the robustness of the conclusions. Additionally, there were too few follow‐up measurements to adequately capture long‐term effects, which is particularly relevant for a population with ongoing health conditions or disabilities. Second, the majority of the studies were conducted in the United States, where case management practices may differ from those in other regions, limiting the generalizability of the findings. Third, not all impairments were represented in the child populations, reducing the relevance to a broader range of chronic conditions as meaningful differences may occur depending on the underlying condition. Most studies relied on self‐reported outcome measures, which, while practical, may introduce bias in the results. Moreover, there was a high risk of bias in the included studies, primarily due to the reliance on self‐reported measures to assess variables and outcomes.

## 5 Conclusion

This systematic review and meta‐analysis provide evidence that case management interventions can offer small to moderate but meaningful improvements for caregivers of children with chronic health conditions, particularly by promoting well‐being and reducing mental distress. The limited number of studies, especially those including follow‐up data and child outcomes, underscores the necessity for further research. Future studies should focus on examining a broader range of chronic conditions, standardizing outcome assessments, and exploring the long‐term effects of case management interventions to allow for more robust and generalizable conclusions.

### 5.1 Relevance for Clinical Practice

Case management interventions can improve the well‐being of caregivers of chronically ill children and reduce their psychological distress, providing a useful adjunct to caregiver support strategies. By incorporating case management into clinical practice, healthcare professionals can provide structured support to carers, addressing their emotional, practical, and logistical challenges. This approach may improve carers' satisfaction with health services and their ability to navigate complex care systems. In addition, improving caregiver support through case management could indirectly benefit the children they care for by fostering a more stable and supportive caregiving environment.

## Author Contributions

**Jan Broll:** conceptualization, methodology, writing – original draft, visualization, data curation, project administration, formal analysis. **Sarah K. Schäfer:** conceptualization, methodology, writing – review and editing, supervision, formal analysis. **Jutta Stoffers‐Winterling:** conceptualization, writing – review and editing. **Sarah Hölzen:** data curation, writing – review and editing. **Isabella Helmreich:** funding acquisition, writing – review and editing, conceptualization. **Klaus Lieb:** conceptualization, funding acquisition, writing – review and editing.

## Ethics Statement

The authors have nothing to report.

## Conflicts of Interest

Three of the authors (J.B., S.K.S., I.H.) were involved in the evaluation of a case management intervention aimed at supporting parents of chronically ill children. The findings of this intervention had not been published at the time of this review and, therefore, could not be included in the analysis. The authors declare that this involvement does not influence the objectivity or validity of the present study.

## Supporting information


**Data S1.** Supporting Information.

## Data Availability

The data that support the findings of this study are openly available in OSF‐Repository at https://osf.io/p47xf/, reference number 10.17605/OSF.IO/P47XF.
