# Supplementary material for: Case Management for Enhancing Wellbeing, Resilience, and Quality of Life in Caregivers of Children and Adolescents With Chronic Illnesses and Disabilities: A Systematic Review and Meta‐Analysis
Source: Nurs Health Sci. 2025 May 8;27(2):e70096. doi: 10.1111/nhs.70096 (PMC12061487; doi:10.1111/nhs.70096)
Supplement: Supplementary file 1 — Data S1. Supporting Information. [file NHS-27-e70096-s001.docx]

**Supplementary Material**

related to ‘**Case Management for Enhancing Wellbeing, Resilience, and Quality of Life in Caregivers of Chronically Ill and Disabled Children and Adolescents: A Systematic Review and Meta-Analysis’**

Table of contents

[Supplementary Data 1. Differences between protocol and review (PROSPERO ID: anonymized for peer review) 2](#_Toc188912902)

[Supplementary Data 2. Search strategies per database 5](#_Toc188912903)

[Supplementary Data 3. Risk of bias ratings 12](#_Toc188912904)

[Supplementary Data 4. GRADE ratings at post-intervention assessments 18](#_Toc188912905)

[Supplementary Data 5. References of studies included in this review 20](#_Toc188912906)

# Supplementary Data 1. Differences between protocol and review (PROSPERO ID: anonymized for peer review)

**Table 1** Differences between protocol and final review

|  | **Protocol** | **Final Review** |
| --- | --- | --- |
| Review design |  | no changes |
| Review question |  | no changes |
| Searches and search strategies |  | no changes |
| Types of included studies |  | no changes |
| Eligible outcomes | - Mental distress (e.g., general mental distress, depressive symptoms, anxiety symptoms, posttraumatic stress symptoms, burnout symptoms) of caregivers and children - Positive mental health (e.g., wellbeing, life satisfaction, [health related] quality of life, vitality, vigour, social relationships/ social support) of caregivers and children - Caregiver burden: Evaluating the perceived burden of caregiving responsibilities on caregivers' emotional, physical, and financial well-being - Healthcare utilization: Tracking the frequency and type of healthcare services accessed by the child and family | Healthcare utilization, originally planned as an outcome in the protocol, was replaced with satisfaction with healthcare services due to difficulties in interpreting healthcare utilization data. This adjustment allowed for a clearer assessment of caregivers' experiences with the healthcare system. |
| Screening and data extraction |  | no changes |
| Risk of bias (quality) assessment |  | no changes |
| Data synthesis |  | no changes |
| Assessment of heterogeneity |  | no changes |
| Subgroup analyses | - Mean sample age of caregivers - Mean sample age of children - Type of caregiver (i.e., mothers, fathers vs. other caregiving persons) - Economic characteristics (e.g., educational level, income) of caregivers - Health status of child - Type of recruitment (e.g., recruited from care/treatment as usual, people voluntarily contracting the study team) - Group vs. individual setting - Degree of parent/caregiver involvement - Intervention providers (e.g., professionals vs. lay persons) - Face-to-face vs. digital intervention (i.e., online or mobile-based) - Intervention intensity (e.g., number and duration of sessions) - Indicators of attrition and compliance - High- vs. middle- vs. low-income contexts - Cultural aspects |  |
| Assessment of publication bias |  | no changes |
| Sensitivity analyses |  | no changes |
|  |  |  |
|  |  |  |

# Supplementary Data 2. Search strategies per database

**Search strategy for primary studies**

**1. PubMed including MEDLINE**

| **#** | **Query** |
| --- | --- |
| 1 | ((((((((Parents[MeSH Terms]) OR (caregivers[MeSH Terms])) OR (Legal Guardians[MeSH Terms])) OR (parent*[Title/Abstract])) OR (caregiver*[Title/Abstract])) OR (mother*[Title/Abstract])) OR (father*[Title/Abstract])) OR (maternal[Title/Abstract])) OR (paternal[Title/Abstract]) |
| 2 | minors[tw] OR boy[tw] OR boys[tw] OR boyhood[tw] OR girl*[tw] OR kid[tw] OR kids[tw] OR child*[tw] OR schoolchild*[tw] OR adolescen*[tw] OR juvenil*[tw] OR youth*[tw] OR teen*[tw] OR preteen*[tw] OR underage*[tw] OR under age*[tw] OR pubescen*[tw] OR paediatric*[tw] OR pediatric*[tw] |
| 3 | ((Home Nursing[MeSH Terms]) OR (Caregivers[MeSH Terms])) OR (Long Term Care[MeSH Terms]) OR (home[Title/Abstract]) OR (home nurs*[Title/Abstract]) |
| 4 | #2 AND #3 |
| 5 | #1 AND #4 |
| 6 | ((((Patient Care Planning[MeSH Terms]) OR (case manage*[Title/Abstract])) ) OR (care plan*[MeSH Terms])) OR (care program*[Title/Abstract]) |
| 7 | (clinical trial[pt] OR comparative study[pt] OR (control[tw] AND study[tw]) OR program[tw] OR epidemiologic studies[mh]) NOT ((animals[mh:noexp] NOT humans[mh:noexp]) OR comment[pt] OR editorial[pt] OR review[pt] OR meta analysis[pt] OR case report [tw] OR consensus[mh] OR guideline[pt] OR history[sh]) |
| 8 | ("randomized controlled trial"[Publication Type] OR "controlled clinical trial"[Publication Type] OR "randomized"[Title/Abstract] OR "placebo"[Title/Abstract] OR "clinical trials as topic"[MeSH Terms:noexp] OR "randomly"[Title/Abstract] OR "trial"[Title]) NOT ("animals"[MeSH Terms] NOT "humans"[MeSH Terms]) |
| 9 | ((clinical trial[pt] OR comparative study[pt] OR (control[tw] AND study[tw]) OR program[tw] OR epidemiologic studies[mh]) NOT ((animals[mh:noexp] NOT humans[mh:noexp]) OR comment[pt] OR editorial[pt] OR review[pt] OR meta analysis[pt] OR case report [tw] OR consensus[mh] OR guideline[pt] OR history[sh])) OR (("randomized controlled trial"[Publication Type] OR "controlled clinical trial"[Publication Type] OR "randomized"[Title/Abstract] OR "placebo"[Title/Abstract] OR "clinical trials as topic"[MeSH Terms:noexp] OR "randomly"[Title/Abstract] OR "trial"[Title]) NOT ("animals"[MeSH Terms] NOT "humans"[MeSH Terms])) |
| 10 | ((#1 AND #4) AND (((((Patient Care Planning[MeSH Terms]) OR (case manage*[Title/Abstract])) ) OR (care plan*[MeSH Terms])) OR (care program*[Title/Abstract]))) AND (((clinical trial[pt] OR comparative study[pt] OR (control[tw] AND study[tw]) OR program[tw] OR epidemiologic studies[mh]) NOT ((animals[mh:noexp] NOT humans[mh:noexp]) OR comment[pt] OR editorial[pt] OR review[pt] OR meta analysis[pt] OR case report [tw] OR consensus[mh] OR guideline[pt] OR history[sh])) OR (("randomized controlled trial"[Publication Type] OR "controlled clinical trial"[Publication Type] OR "randomized"[Title/Abstract] OR "placebo"[Title/Abstract] OR "clinical trials as topic"[MeSH Terms:noexp] OR "randomly"[Title/Abstract] OR "trial"[Title]) NOT ("animals"[MeSH Terms] NOT "humans"[MeSH Terms]))) |
| 11 | ((#1 AND #4) AND (((((Patient Care Planning[MeSH Terms]) OR (case manage*[Title/Abstract])) ) OR (care plan*[MeSH Terms])) OR (care program*[Title/Abstract]))) AND (("randomized controlled trial"[Publication Type] OR "controlled clinical trial"[Publication Type] OR "randomized"[Title/Abstract] OR "placebo"[Title/Abstract] OR "clinical trials as topic"[MeSH Terms:noexp] OR "randomly"[Title/Abstract] OR "trial"[Title]) NOT ("animals"[MeSH Terms] NOT "humans"[MeSH Terms])) |

**2. APA PsycNet (PsycInfo, PsycArticles, PsycExtra)**

| **#** | **Query** |
| --- | --- |
| 1 | abstract: parent* OR abstract: mother* OR abstract: father* OR abstract: maternal OR abstract: paternal OR abstract: caregiver* OR abstract: guardian* OR KEYWORDS: Parents OR KEYWORDS: Caregiver OR KEYWORDS: Guardianship |
| 2 | abstract: minors OR abstract: boy* OR abstract: girl* OR abstract: kid OR abstract: child* OR abstract: schoolchild* OR abstract: adolescen* OR abstract: juvenil* OR abstract: youth OR abstract: underage* OR abstract: pubescen* OR abstract: paediatric* |
| 3 | abstract: Home Care OR abstract: Homecare OR abstract: domiciliary care OR abstract: social care OR abstract: in-home care OR abstract: Long Term Care OR abstract: home OR abstract: home nurs* OR KEYWORDS: Home Care OR KEYWORDS: Homecare OR KEYWORDS: domiciliary care OR KEYWORDS: social care OR KEYWORDS: in-home care OR KEYWORDS: Long Term Care |
| 4 | (abstract: (minors) OR abstract: (boy*) OR abstract: (girl*) OR abstract: (kid) OR abstract: (child*) OR abstract: (schoolchild*) OR abstract: (adolescen*) OR abstract: (juvenil*) OR abstract: (youth) OR abstract: (underage*) OR abstract: (pubescen*) OR abstract: (paediatric*)) AND (abstract: (Home Care) OR abstract: (Homecare) OR abstract: (domiciliary care) OR abstract: (social care) OR abstract: (in-home care) OR abstract: (Long Term Care) OR abstract: (home) OR abstract: (home nurs*) OR KEYWORDS: (Home Care) OR KEYWORDS: (Homecare) OR KEYWORDS: (domiciliary care) OR KEYWORDS: (social care) OR KEYWORDS: (in-home care) OR KEYWORDS: (Long Term Care)) |
| 5 | (abstract: (parent*) OR abstract: (mother*) OR abstract: (father*) OR abstract: (maternal) OR abstract: (paternal) OR abstract: (caregiver*) OR abstract: (guardian*) OR KEYWORDS: (Parents) OR KEYWORDS: (Caregiver) OR KEYWORDS: (Guardianship)) AND (abstract: (minors) OR abstract: (boy*) OR abstract: (girl*) OR abstract: (kid) OR abstract: (child*) OR abstract: (schoolchild*) OR abstract: (adolescen*) OR abstract: (juvenil*) OR abstract: (youth) OR abstract: (underage*) OR abstract: (pubescen*) OR abstract: (paediatric*)) AND (abstract: (Home Care) OR abstract: (Homecare) OR abstract: (domiciliary care) OR abstract: (social care) OR abstract: (in-home care) OR abstract: (Long Term Care) OR abstract: (home) OR abstract: (home nurs*) OR KEYWORDS: (Home Care) OR KEYWORDS: (Homecare) OR KEYWORDS: (domiciliary care) OR KEYWORDS: (social care) OR KEYWORDS: (in-home care) OR KEYWORDS: (Long Term Care)) |
| 6 | abstract: Treatment Planning OR abstract: case manage* OR abstract: care OR abstract: care program OR KEYWORDS: Treatment Planning |
| 7 | abstract: clinical trial OR abstract: comparative study OR abstract: control OR abstract: study OR abstract: program OR KEYWORDS: Epidemiolog |
| 8 | abstract: placebo* OR abstract: random* OR abstract: comparative stud* OR abstract: clinical trial* OR abstract: research design OR abstract: evaluative stud* OR abstract: prospectiv stud* OR KEYWORDS: Treatment Effectiveness Evaluation OR KEYWORDS: Treatment Outcomes OR KEYWORDS: Placebo OR KEYWORDS: Followup Studies |
| 9 | (abstract: (clinical trial) OR abstract: (comparative study) OR abstract: (control) OR abstract: (study) OR abstract: (program) OR KEYWORDS: (Epidemiolog)) OR (abstract: (placebo*) OR abstract: (random*) OR abstract: (comparative stud*) OR abstract: (clinical trial*) OR abstract: (research design) OR abstract: (evaluative stud*) OR abstract: (prospectiv stud*) OR KEYWORDS: (Treatment Effectiveness Evaluation) OR KEYWORDS: (Treatment Outcomes) OR KEYWORDS: (Placebo) OR KEYWORDS: (Followup Studies)) |
| 10 | (((abstract: (parent*)) OR (abstract: (mother*)) OR (abstract: (father*)) OR (abstract: (maternal)) OR (abstract: (paternal)) OR (abstract: (caregiver*)) OR (abstract: (guardian*)) OR (KEYWORDS: (Parents)) OR (KEYWORDS: (Caregiver)) OR (KEYWORDS: (Guardianship))) AND ((abstract: (minors)) OR (abstract: (boy*)) OR (abstract: (girl*)) OR (abstract: (kid)) OR (abstract: (child*)) OR (abstract: (schoolchild*)) OR (abstract: (adolescen*)) OR (abstract: (juvenil*)) OR (abstract: (youth)) OR (abstract: (underage*)) OR (abstract: (pubescen*)) OR (abstract: (paediatric*))) AND ((abstract: (Home Care)) OR (abstract: (Homecare)) OR (abstract: (domiciliary care)) OR (abstract: (social care)) OR (abstract: (in-home care)) OR (abstract: (Long Term Care)) OR (abstract: (home)) OR (abstract: (home nurs*)) OR (KEYWORDS: (Home Care)) OR (KEYWORDS: (Homecare)) OR (KEYWORDS: (domiciliary care)) OR (KEYWORDS: (social care)) OR (KEYWORDS: (in-home care)) OR (KEYWORDS: (Long Term Care)))) AND (abstract: (Treatment Planning) OR abstract: (case manage*) OR abstract: (care) OR abstract: (care program) OR KEYWORDS: (Treatment Planning)) AND (abstract: (placebo*) OR abstract: (random*) OR abstract: (comparative stud*) OR abstract: (clinical trial*) OR abstract: (research design) OR abstract: (evaluative stud*) OR abstract: (prospectiv stud*) OR KEYWORDS: (Treatment Effectiveness Evaluation) OR KEYWORDS: (Treatment Outcomes) OR KEYWORDS: (Placebo) OR KEYWORDS: (Followup Studies)) |
| 11 | (((abstract: (parent*)) OR (abstract: (mother*)) OR (abstract: (father*)) OR (abstract: (maternal)) OR (abstract: (paternal)) OR (abstract: (caregiver*)) OR (abstract: (guardian*)) OR (KEYWORDS: (Parents)) OR (KEYWORDS: (Caregiver)) OR (KEYWORDS: (Guardianship))) AND ((abstract: (minors)) OR (abstract: (boy*)) OR (abstract: (girl*)) OR (abstract: (kid)) OR (abstract: (child*)) OR (abstract: (schoolchild*)) OR (abstract: (adolescen*)) OR (abstract: (juvenil*)) OR (abstract: (youth)) OR (abstract: (underage*)) OR (abstract: (pubescen*)) OR (abstract: (paediatric*))) AND ((abstract: (Home Care)) OR (abstract: (Homecare)) OR (abstract: (domiciliary care)) OR (abstract: (social care)) OR (abstract: (in-home care)) OR (abstract: (Long Term Care)) OR (abstract: (home)) OR (abstract: (home nurs*)) OR (KEYWORDS: (Home Care)) OR (KEYWORDS: (Homecare)) OR (KEYWORDS: (domiciliary care)) OR (KEYWORDS: (social care)) OR (KEYWORDS: (in-home care)) OR (KEYWORDS: (Long Term Care)))) AND (abstract: (Treatment Planning) OR abstract: (case manage*) OR abstract: (care) OR abstract: (care program) OR KEYWORDS: (Treatment Planning)) AND (abstract: (placebo*) OR abstract: (random*) OR abstract: (comparative stud*) OR abstract: (clinical trial*) OR abstract: (research design) OR abstract: (evaluative stud*) OR abstract: (prospectiv stud*) OR KEYWORDS: (Treatment Effectiveness Evaluation) OR KEYWORDS: (Treatment Outcomes) OR KEYWORDS: (Placebo) OR KEYWORDS: (Followup Studies)) |

**3. Cochrane Central Register of Controlled Trials (CENTRAL)**

| **#** | **Query** |
| --- | --- |
| 1 | parent* in Title Abstract Keyword OR mother* in Title Abstract Keyword OR father* in Title Abstract Keyword OR caregiver* in Title Abstract Keyword OR guardian* in Title Abstract Keyword |
| 2 | child* in Title Abstract Keyword OR adolescen* in Title Abstract Keyword OR juvenil* in Title Abstract Keyword OR underage* in Title Abstract Keyword OR paediatric* in Title Abstract Keyword |
| 3 | home nurs* in Title Abstract Keyword OR Homecare in Title Abstract Keyword OR in-home care in Title Abstract Keyword OR domiciliary care in Title Abstract Keyword OR home* in Title Abstract Keyword |
| 4 | #2 AND #3 |
| 5 | #1 AND #2 AND #3 |
| 6 | Treatment Planning in Abstract OR case manage* in Abstract OR care in Abstract OR care program in Abstract OR Treatment Planning in Title Abstract Keyword |
| 7 | #1 AND #2 AND #3 AND #6 |

**4. Scopus.com**

| **#** | **Query** |
| --- | --- |
| 1 | ((((((((((AB=(parent* )) OR AB=(mother* ))) OR AB=(father*)) OR AB=(maternal)) OR AB=(paternal )) OR AB=(caregiver*)) OR AB=(guardian*)) OR TS=(Parents )) OR TS=(Caregiver)) OR TS=(Guardianship) |
| 2 | (((((((((((AB=(minors)) OR AB=(boy* )) OR AB=(girl* )) OR AB=(kid)) OR AB=(child* )) OR AB=(schoolchild* )) OR AB=(adolescen* )) OR AB=(juvenil* )) OR AB=(youth)) OR AB=(underage* )) OR AB=(pubescen* )) OR AB=(paediatric*) |
| 3 | ((((((AB=(home nurs*)) OR AB=(Home Care)) OR AB=(Caregivers)) OR AB=(Long Term Care)) OR TS=(Long Term Care)) OR TS=(Home Care)) OR TS=(Caregivers) |
| 4 | #2 AND #3 |
| 5 | #1 AND #2 AND #3 |
| 6 | ((AB=(case manage* )) OR AB=(care program)) OR TS=(Treatment Planning) |
| 7 | (((((AB=(clinical trial)) OR AB=(comparative study )) OR AB=(control)) OR AB=(study)) OR AB=(program)) OR AB=(Epidemiolog*) |
| 8 | ((((((((((AB=(placebo* )) OR AB=(random* )) OR AB=( comparative stud* )) OR AB=( clinical trial* )) OR AB=(research design )) OR AB=(evaluative stud* )) OR AB=(prospectiv stud* )) OR TS=(Treatment Effectiveness Evaluation )) OR TS=(Treatment Outcomes )) OR TS=(Placebo)) OR TS=(Followup Studies) |
| 9 | #7 OR #8 |
| 10 | #5 AND #6 AND #9 |
| 11 | #5 AND #6 AND #8 |

**5. Web of Science**

| **#** | **Query** |
| --- | --- |
| 1 | ((((((((((AB=(parent* )) OR AB=(mother* ))) OR AB=(father*)) OR AB=(maternal)) OR AB=(paternal )) OR AB=(caregiver*)) OR AB=(guardian*)) OR TS=(Parents )) OR TS=(Caregiver)) OR TS=(Guardianship) |
| 2 | (((((((((((AB=(minors)) OR AB=(boy* )) OR AB=(girl* )) OR AB=(kid)) OR AB=(child* )) OR AB=(schoolchild* )) OR AB=(adolescen* )) OR AB=(juvenil* )) OR AB=(youth)) OR AB=(underage* )) OR AB=(pubescen* )) OR AB=(paediatric*) |
| 3 | ((((((AB=(home nurs*)) OR AB=(Home Care)) OR AB=(Caregivers)) OR AB=(Long Term Care)) OR TS=(Long Term Care)) OR TS=(Home Care)) OR TS=(Caregivers) |
| 4 | #2 AND #3 |
| 5 | #1 AND #2 AND #3 |
| 6 | ((AB=(case manage* )) OR AB=(care program)) OR TS=(Treatment Planning) |
| 7 | (((((AB=(clinical trial)) OR AB=(comparative study )) OR AB=(control)) OR AB=(study)) OR AB=(program)) OR AB=(Epidemiolog*) |
| 8 | ((((((((((AB=(placebo* )) OR AB=(random* )) OR AB=( comparative stud* )) OR AB=( clinical trial* )) OR AB=(research design )) OR AB=(evaluative stud* )) OR AB=(prospectiv stud* )) OR TS=(Treatment Effectiveness Evaluation )) OR TS=(Treatment Outcomes )) OR TS=(Placebo)) OR TS=(Followup Studies) |
| 9 | #7 OR #8 |
| 10 | #5 AND #6 AND #9 |
| 11 | #5 AND #6 AND #8 |

# Supplementary Data 3. Risk of bias ratings

**Table 2** Risk of bias for included effect estimates based on the Cochrane risk-of-bias tool for randomized trials (RoB2)

| Study | D1: Randomization Process | D2: Deviations from Intervention | D3: Missing Outcome Data | D4: Outcome Measurement | D5: Selection of Reported Result |
| --- | --- | --- | --- | --- | --- |
| Badr 2006a | Low | Some concerns | Some concerns | High | Low |
| Badr 2006b | Low | Some concerns | Some concerns | High | Low |
| Badr 2006c | Low | Some concerns | Some concerns | High | Low |
| Bernie 2023a | Low | Low | Low | High | Low |
| Bernie 2023b | Low | Low | Low | High | Low |
| Bernie 2023c | Low | Low | Low | High | Low |
| Bernie 2023d | Low | Low | Low | High | Low |
| Bilgin 2009a | Low | Low | Low | High | Some concerns |
| Bilgin 2009b | Low | Low | Low | High | Some concerns |
| Burton 2018a | Low | Low | Some concerns | High | Some concerns |
| Burton 2018b | Low | Low | Some concerns | High | Some concerns |
| Burton 2018c | Low | Low | Some concerns | High | Some concerns |
| Burton 2018d | Low | Low | Some concerns | High | Some concerns |
| Cady 2015a | Low | Some concerns | Some concerns | High | Low |
| Cady 2015b | Low | Some concerns | Some concerns | High | Low |
| Cady 2015c | Low | Some concerns | Some concerns | High | Low |
| Chan 2007a | Some concerns | Some concerns | High | High | Low |
| Chan 2007b | Some concerns | Some concerns | High | High | Low |
| Chen 2013a | High | Low | Some concerns | High | Low |
| Chen 2013b | High | Low | Some concerns | High | Low |
| Cohen 2023a | Low | Low | Low | High | Low |
| Cohen 2023b | Low | Low | Low | High | Low |
| Cohen 2023c | Low | Low | Low | High | Low |
| Cohen 2023d | Low | Low | Low | High | Low |
| Cohen 2023e | Low | Low | Low | High | Low |
| Cohen 2023f | Low | Low | Low | High | Low |
| Cohen 2023g | Low | Low | Low | High | Low |
| Cohen 2023h | Low | Low | Low | High | Low |
| Farmer 2011a | Some concerns | Low | Low | High | Low |
| Farmer 2011b | Some concerns | Low | Low | High | Low |
| Farmer 2011c | Some concerns | Low | Low | High | Low |
| Flores 2009a | Low | Low | Some concerns | High | Low |
| Flores 2009b | Low | Low | Some concerns | High | Low |
| Flores 2009c | Low | Low | Some concerns | High | Low |
| Liu 2020a | Some concerns | Some concerns | High | High | Low |
| Liu 2020b | Some concerns | Some concerns | High | High | Low |
| Liu 2020c | Some concerns | Some concerns | High | High | Low |
| Liu 2020d | Some concerns | Some concerns | High | High | Low |
| Liu 2020e | Some concerns | Some concerns | High | High | Low |
| Looman 2015a | Some concerns | Some concerns | Low | High | Low |
| Looman 2015b | Some concerns | Some concerns | Low | High | Low |
| Looman 2015c | Some concerns | Some concerns | Low | High | Low |
| Looman 2015d | Some concerns | Some concerns | Low | High | Low |
| Looman 2015e | Some concerns | Some concerns | Low | High | Low |
| Looman 2015f | Some concerns | Some concerns | Low | High | Low |
| Looman 2015g | Some concerns | Some concerns | Low | High | Low |
| Looman 2015h | Some concerns | Some concerns | Low | High | Low |
| Looman 2018a | Some concerns | Some concerns | Low | High | Low |
| Looman 2018b | Some concerns | Some concerns | Low | High | Low |
| Looman 2018c | Some concerns | Some concerns | Low | High | Low |
| Looman 2018d | Some concerns | Some concerns | Low | High | Low |
| Looman 2018e | Some concerns | Some concerns | Low | High | Low |
| Looman 2018f | Some concerns | Some concerns | Low | High | Low |
| Looman 2018g | Some concerns | Some concerns | Low | High | Low |
| Looman 2018h | Some concerns | Some concerns | Low | High | Low |
| Moody 2019 | Some concerns | Low | Low | High | Low |
| Seid 2010a | Low | Low | Some concerns | High | Low |
| Seid 2010b | Low | Low | Some concerns | High | Low |
| Thompkins 2021a | Low | Low | Low | High | High |
| Thompkins 2021b | Low | Low | Low | High | High |
| Thompkins 2021c | Low | Low | Low | High | High |
| Zhang 2023a | Low | Some concerns | Low | High | Low |
| Zhang 2023b | Low | Some concerns | Low | High | Low |
| Zhang 2023c | Low | Some concerns | Low | High | Low |

# Supplementary Data 4. GRADE ratings at post-intervention assessments

**Table 3** GRADE assessment for positive mental health (post assessment)

| **GRADE criteria** | **Rating** | **Notes** | **Certainty of evidence** |
| --- | --- | --- | --- |
| **Study design** | **RCT (high quality)** | All of included studies were RCTs, contributing to high quality. | ⊕⊕◯◯  low |
| **Risk of Bias**  (Cochrane Risk of Bias) | serious (-1) | High risk of bias due to reliance on self-reported outcome measures and lack of blinding for most studies. |  |
| **Inconsistency** | **Not serious** | Low heterogeneity observed *(I*² = 10.8%), indicating consistent findings across studies. |  |
| **Indirectness** | **serious (-1)** | Studies primarily conducted in high-income countries and focused on specific populations, limiting generalizability. |  |
| **Imprecision** | **Not serious** | Confidence intervals were narrow, and the sample size was adequate to detect small effects. |  |
| **Publication Bias** | Undetected | Funnel plot symmetry and rank correlation test indicated no evidence of publication bias for positive mental health. |  |
| **Other**  (upgrading factor) | None | No evidence for large effects, dose-response relationships, or plausible confounding that would increase confidence. |  |

**Source**: Modified after Ryan R, Hill S (2016) How to GRADE the quality of the evidence. Cochrane Consumers and Communication Group, available at http://cccrg.cochrane.org/author-resources.

**Table 4** GRADE assessment for mental distress (post assessment)

| **GRADE criteria** | **Rating** | **Notes** | **Certainty of evidence** |
| --- | --- | --- | --- |
| **Study design** | **RCT (high quality)** | All of included studies were RCTs, contributing to high quality. | ⊕⊕◯◯  low |
| **Risk of Bias**  (Cochrane Risk of Bias) | serious (-1) | High risk of bias due to reliance on self-reported outcome measures and lack of blinding for most studies. |  |
| **Inconsistency** | **Not serious** | Low heterogeneity observed (*I*² = 2.7%), indicating consistent findings across studies. |  |
| **Indirectness** | **serious (-1)** | Studies primarily conducted in high-income countries and focused on specific populations, limiting generalizability. |  |
| **Imprecision** | **Not serious** | Confidence intervals were relatively narrow, and the sample size was adequate to detect small to moderate effects. |  |
| **Publication Bias** | Undetected | Funnel plot symmetry and rank correlation test indicated no evidence of publication bias for mental distress outcomes. |  |
| **Other**  (upgrading factor) | None | No evidence for large effects, dose-response relationships, or plausible confounding that would increase confidence. |  |

**Source**: Modified after Ryan R, Hill S (2016) How to GRADE the quality of the evidence. Cochrane Consumers and Communication Group, available at http://cccrg.cochrane.org/author-resources.

**Table 5** GRADE assessment for satisfaction with healthcare services (post assessment)

| **GRADE criteria** | **Rating** | **Notes** | **Certainty of evidence** |
| --- | --- | --- | --- |
| **Study design** | **RCT (high quality)** | All of included studies were RCTs, contributing to high quality. | ⊕◯◯◯  Very low |
| **Risk of Bias**  (Cochrane Risk of Bias) | serious (-1) | High risk of bias due to reliance on self-reported outcome measures and lack of blinding for most studies. |  |
| **Inconsistency** | **serious (-1)** | Slight asymmetry in the funnel plot and variability in intervention effects observed across studies. |  |
| **Indirectness** | **serious (-1)** | Studies primarily conducted in high-income countries and focused on specific populations, limiting generalizability. |  |
| **Imprecision** | **Not serious** | Confidence intervals were reasonably narrow, and sample size was adequate to detect small effects |  |
| **Publication Bias** | Undetected | Although the funnel plot showed slight asymmetry, further inspection did not indicate a significant risk of publication bias. |  |
| **Other**  (upgrading factor) | None | No evidence for large effects, dose-response relationships, or plausible confounding that would increase confidence. |  |

**Source**: Modified after Ryan R, Hill S (2016) How to GRADE the quality of the evidence. Cochrane Consumers and Communication Group, available at http://cccrg.cochrane.org/author-resources.

# Supplementary Data 5. References of studies included in this review

Badr, L. K., Garg, M., & Kamath, M. (2006). Intervention for infants with brain injury: Results of a randomized controlled study. *Infant Behavior & Development, 29*(1), 80–90. psyh. https://doi.org/10.1016/j.infbeh.2005.08.003

Bernie, C., Williams, K., Graham, F., & May, T. (2023). Coaching while waiting for autism spectrum disorder assessment: A pilot feasibility study for a randomized controlled trial on occupational performance coaching and service navigation. *Journal of Autism and Developmental Disorders, 53*(7), 2905–2914. https://doi.org/10.1007/s10803-022-05558-3

Bilgin, S., & Gozum, S. (2009). Reducing burnout in mothers with an intellectually disabled child: An education programme. *Journal of Advanced Nursing, 65*(12), 2552–5261. https://doi.org/10.1111/j.1365-2648.2009.05163.x

Burton, R. S., Zwahr-Castro, J., Magrane, C. L., Hernandez, H., Farley, L. G., & Amodei, N. (2018). The nurturing program: An intervention for parents of children with special needs. *Journal of Child and Family Studies, 27*(4), 1137–1149. https://doi.org/10.1007/s10826-017-0966-3

Cady, R. G., Erickson, M., Lunos, S., Finkelstein, S. M., Looman, W., Celebreeze, M., & Garwick, A. (2015). Meeting the needs of children with medical complexity using a telehealth advanced practice registered nurse care coordination model. *Maternal and Child Health Journal, 19*(7), 1497–1506. https://doi.org/10.1007/s10995-014-1654-1

Chan, D. S., Callahan, C. W., Hatch-Pigott, V. B., Lawless, A., Proffitt, H. L., Manning, N. E., Schweikert, M., & Malone, F. J. (2007). Internet-based home monitoring and education of children with asthma is comparable to ideal office-based care: Results of a 1-year asthma in-home monitoring trial. *Pediatrics, 119*(3), 569–578. https://doi.org/10.1542/peds.2006-1884

Chen, S.-H., Huang, J.-L., Yeh, K.-W., & Tsai, Y.-F. (2013). Interactive support interventions for caregivers of asthmatic children. *The Journal of Asthma, 50*(6), 649–657. https://doi.org/10.3109/02770903.2013.794236

Cohen, E., Quartarone, S., Orkin, J., Moretti, M. E., Emdin, A., Guttmann, A., Willan, A. R., Major, N., Lim, A., Diaz, S., Osqui, L., Soscia, J., Fu, L., Gandhi, S., Heath, A., & Fayed, N. (2023). Effectiveness of structured care coordination for children with medical complexity: The complex care for kids ontario (CCKO) randomized clinical trial. *JAMA Pediatrics, 177*(5), 461–471. https://doi.org/10.1001/jamapediatrics.2023.0115

Farmer, J. E., Clark, M. J., Drewel, E. H., Swenson, T. M., & Ge, B. (2011). Consultative care coordination through the medical home for CSHCN: A randomized controlled trial. *Maternal and Child Health Journal, 15*(7), 1110–1118. https://doi.org/10.1007/s10995-010-0658-8

Flores, G., Bridon, C., Torres, S., Perez, R., Walter, T., Brotanek, J., Lin, H., & Tomany-Korman, S. (2009). Improving Asthma Outcomes in Minority Children: A Randomized, Controlled Trial of Parent Mentors. *Pediatrics, 124*(6), 1522–1532. https://doi.org/10.1542/peds.2009-0230

Liu, H., Song, Q., Zhu, L., Chen, D., Xie, J., Hu, S., Zeng, S., & Tan, L. (2020). Family management style improves family quality of life in children with epilepsy: A randomized controlled trial. Journal of Neuroscience *Nursing, 52*(2), 84. https://doi.org/10.1097/JNN.0000000000000497

Looman, W. S., Antolick, M., Cady, R. G., Lunos, S. A., Garwick, A. E., & Finkelstein, S. M. (2015). Effects of a telehealth care coordination intervention on perceptions of health care by caregivers of children with medical complexity: A randomized controlled trial. *Journal of Pediatric Health Care, 29*(4), 352–363. https://doi.org/10.1016/j.pedhc.2015.01.007

Looman, W. S., Hullsiek, R. L., Pryor, L., Mathiason, M. A., & Finkelstein, S. M. (2018). Health-related quality of life outcomes of a telehealth care coordination intervention for children with medical complexity: A randomized controlled trial. *Journal of Pediatric Health Care, 32*(1), 63–75. https://doi.org/10.1016/j.pedhc.2017.07.007

Moody, E. J., Kaiser, K., Sharp, D., Kubicek, L. F., Rigles, B., Davis, J., McSwegin, S., D’Abreu, L. C., & Rosenberg, C. R. (2019). Improving family functioning following diagnosis of ASD: A randomized trial of a parent mentorship program. *Journal of Child and Family Studies, 28*(2), 424–435. https://doi.org/10.1007/s10826-018-1293-z

Seid, M., Varni, J. W., Gidwani, P., Gelhard, L. R., & Slymen, D. J. (2010). Problem-solving skills training for vulnerable families of children with persistent asthma: Report of a randomized trial on health-related quality of life outcomes. *Journal of Pediatric Psychology, 35*(10), 1133–1143. https://doi.org/10.1093/jpepsy/jsp133

Thompkins, J. D., Needle, J., Baker, J. N., Briggs, L., Cheng, Y. I., Wang, J., Friebert, S., & Lyon, M. E. (2021). Pediatric advance care planning and families’ positive caregiving appraisals: An RCT. *Pediatrics, 147*(6), e2020029330. https://doi.org/10.1542/peds.2020-029330

Zhang, Q.-L., Lin, S.-H., Lin, W.-H., Chen, Q., & Cao, H. (2023). The effect of applying telehealth education to home care of infants after congenital heart disease surgery. *International Journal for Quality in Health Care, 35*(1), mzac102. https://doi.org/10.1093/intqhc/mzac102
